# Supplementary material for: Chromothripsis during telomere crisis is independent of NHEJ, and consistent with a replicative origin
Source: Genome Res. 2019 May;29(5):737–49. doi: 10.1101/gr.240705.118 (PMC6499312; doi:10.1101/gr.240705.118)
Supplement: Supplemental Material [file supp_gr.240705.118_Supplemental_file_1.zip › contigs/annotated_contigs/DB48/contig.2.DB48_length_742_mean_cov_7.45552560647.docx]

**DB48_length_742_mean_cov_7.45552560647**

```
CTTTGTGAAAGAGCTGAAACTCTTTCCTCAGAGTTTCAAAAGAAGCCATGTCAAGTCCGAGTGAAGTGTGACTCTTGGAGGAAAAAGAA
 >chr10:109899225-109899567 - E=6e-194
AAACATGATATGCTAGGGTATTGATGATGAGGGGCAGTTAATCCTTCGGGATTTGAGTGTATTTCAGAGAGTAAGAACACAGTTGCAGT

CAGCCCTGTTTCTACTGGCAACATTTCTTTCCATCTATTACCTACACCCATCCATGACATCTGACATTCACCTCACAAGGCCGAATGGC

TTAGTTCTCTGGAAATTTCTAAGTGTCAGCTTTTAATATAGTATGCTTCGAGGTTTAAAATCATCTTCAGCCAAA|TTCCAGGGAAGCC
                                                                             >chr10:1098
TGCACTTGGTCACTAAGAAGGACAGAGTTATAGAGGCTTGGTTAATCAGGATCCTTTGACGGGAAATCTTCACTCCAAAGGTCTCCACC
96761-109897164 - E=2e-220
TTGCTGGCTGAGGCTTTGTCAGGCTTGCATCACATTTCTGCTTTTTCCTCTACCTAATTTTGTTTCTCTTCCTTTTCTTTTCTTTCGGA

GATGTTAATCCCTAGCCAGTATCTTACACTTCAGATACCATCTCAGCTTTTCTTTCTTTCTTCTTCTTTTTTTTTTTCAATTCCTGGAT

GGCCCAAATTGCTACAGACAGCAATAAAAATGCAGTGGTTTGAGATACATGTAATGAGATGAGATTTTGGAGCTGGATCAAACACTTTC

TTGCTGGCAATGAGTACTCCTTCACTGGTGG
```
